# Uncommon Sequela of Miscarriage: A Case of Hematometra

**DOI:** 10.7759/cureus.89288

**Published:** 2025-08-03

**Authors:** Jaice M Devasia, Reshma Rafi

**Affiliations:** 1 Obstetrics and Gynaecology, Whittington Health National Health Service (NHS) Trust, London, GBR

**Keywords:** complication, hematometra, miscarriage, suction and evacuation, ultrasound

## Abstract

Hematometra is an uncommon and delayed complication following the surgical management of miscarriage. When it occurs in the early postoperative period, it is sometimes referred to as "Redo syndrome". We present the case of a 39-year-old woman who developed this rare condition following a suction and evacuation procedure performed for a missed miscarriage. Within 48 hours of the procedure, the patient presented with acute lower abdominal pain, low-grade fever, dysuria, and pain during defecation. Despite her symptoms, her vital signs remained stable. However, laboratory investigations revealed an elevated C-reactive protein (CRP) level, indicating inflammation. She was started on broad-spectrum antibiotics. A transvaginal ultrasound revealed the presence of hematometra, a collection of blood within the uterine cavity. The patient underwent a repeat uterine evacuation under ultrasound guidance, during which approximately 50 mL of dark blood clots were removed. She recovered well post-procedure and was discharged the following day in a stable condition. Follow-up scan done after two weeks was normal. This case highlights the importance of considering hematometra, or Redo Syndrome, in patients presenting with abdominal pain and systemic symptoms shortly after surgical miscarriage management. Early recognition and prompt intervention are essential to prevent further complications and to ensure patient recovery.

## Introduction

Accumulation of blood in the uterus, called hematometra, is a rare complication of management of miscarriage. This condition is called the Redo syndrome [1]. The incidence of Redo syndrome is 0.1-1 per 100 cases of suction and evacuation [1]. Redo syndrome can also occur after any uterine procedures like caesarean section [2]. Despite its low frequency, its potential for causing considerable patient morbidity underscores the importance of the early recognition and management of this condition. Hematometra typically arises due to cervical stenosis, which may result from congenital anomalies or be acquired following surgical interventions such as dilation and curettage or cervical cautery. The narrowed cervical canal impedes normal menstrual outflow, leading to blood accumulation within the uterus. The clinical symptoms of hematometra include amenorrhea, pelvic pain, rectal tenesmus and retention of urine [3]. If the retained blood becomes infected, systemic symptoms such as fever may develop, further complicating the clinical picture [3]. Clinical examination can reveal enlarged tender uterus due to the collection of blood. Transvaginal sonography usually aids in the diagnosis. Prompt evacuation of the uterus is the proposed management. Timely diagnosis is vital, as untreated hematometra can lead to serious complications, including pyometra, peritonitis, endometriosis, pelvic inflammatory disease and sepsis [4]. Given the nonspecific presentation and potential for diagnostic delay, it is imperative for clinicians to maintain a high index of suspicion in patients presenting with post-abortal pain or amenorrhea. Prompt uterine evacuation remains the mainstay of treatment, offering both symptom relief and prevention of further complications. Hysteroscopic adhesiolysis and cervical dilatation can be performed if there is recurrence of hematometra [5]. This case report highlights a rare instance of post-abortal hematometra, with an emphasis on the diagnostic challenges and clinical implications of this under-recognized condition.

## Case presentation

This is the case of a 39-year-old woman, who conceived through in vitro fertilization in the context of a same-sex couple. She underwent surgical management for early miscarriage, with an estimated blood loss of 250 mL during the procedure, which was otherwise uneventful.

Within 48 hours post-procedure, she presented to the emergency department with worsening lower abdominal pain, described as "contraction-type," radiating to the inner aspect of her right thigh. She also reported low-grade fever, pain during urination, and pain while opening her bowels. However, her vaginal bleeding was reported to be normal, with no accompanying foul-smelling discharge.

Upon review, she appeared distressed due to pain. Her general observations were within normal limits. Abdominal examination revealed significant tenderness in the lower abdomen with voluntary guarding. Speculum examination was unremarkable, while bimanual examination highlighted bilateral forniceal tenderness and notable uterine tenderness. The uterus was observed to be bulky and mobile.

**Figure 1:**
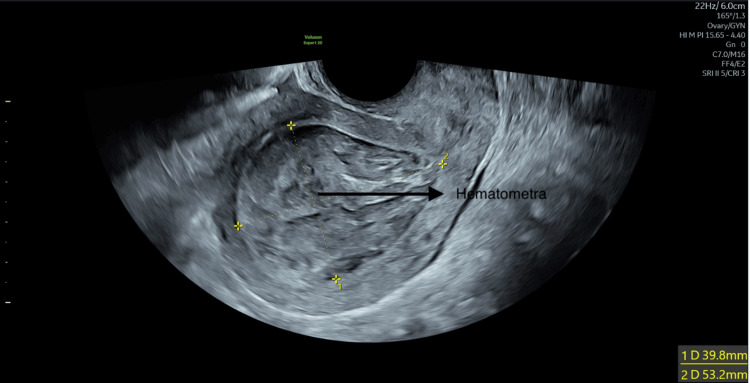
Uterine cavity distended with blood

Her hemoglobin level was 126 g/L and C-reactive protein (CRP) was elevated at 46 mg/L. The initial differential diagnoses included endometritis and uterine perforation. Intravenous antibiotics were initiated promptly. A transvaginal ultrasonography revealed hematometra measuring 53 mm×50 mm×40 mm distending the uterine cavity (Figure 1).

**Figure 2:**
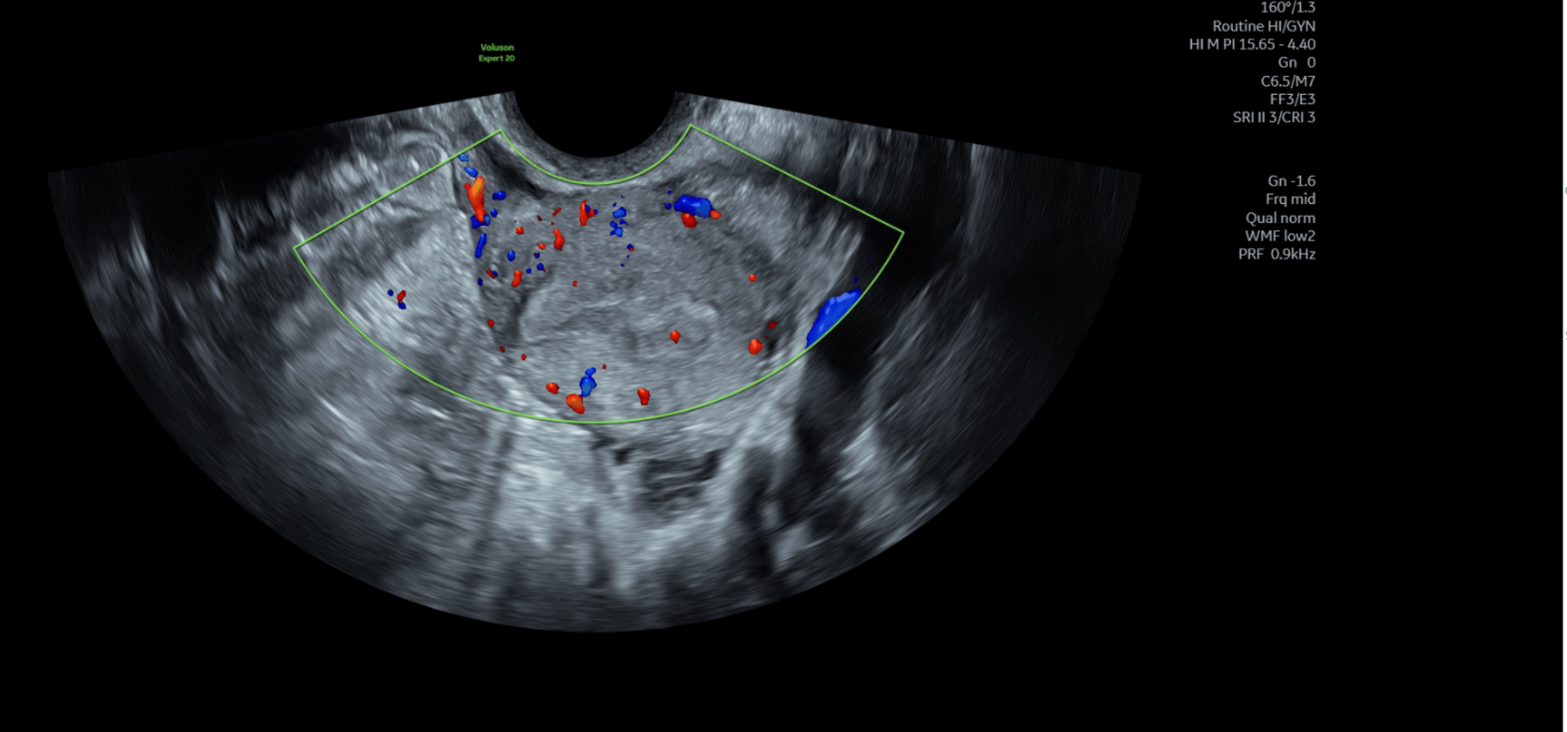
No vascularity inside the endometrium confirming hematometra. Normal vascularity in the myometrium

There was no vascularity, confirming it to be hematometra (Figure 2). This led to the decision for the evacuation of hematometra under general anesthesia.

**Figure 3:**
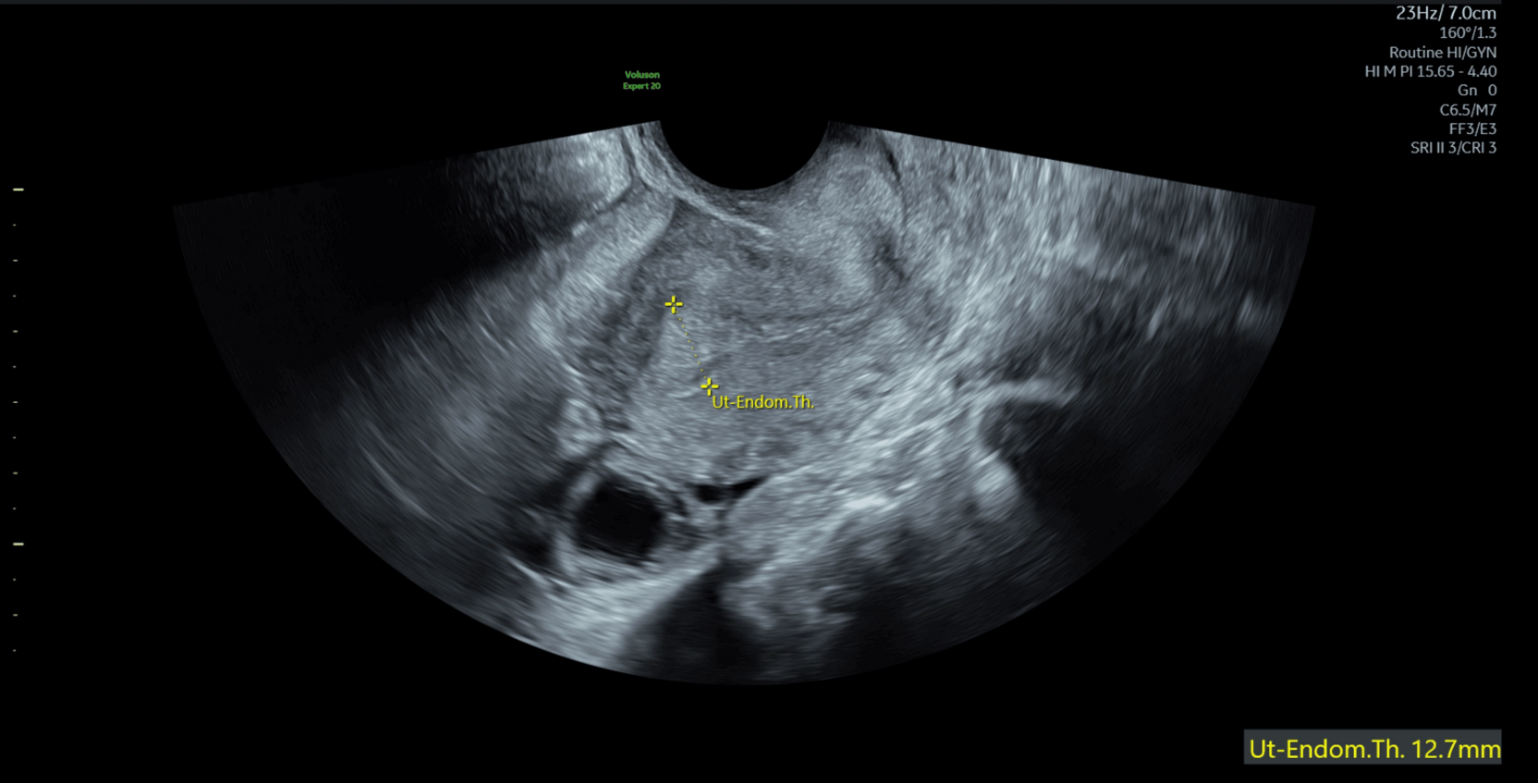
Normal endometrium

During the procedure, the internal os was tightly closed and required dilation to Hegar dilator size 8. Approximately 50 mL of dark blood was evacuated under ultrasound guidance, with no evidence of retained products of conception observed. The patient was monitored for 24 hours and subsequently discharged. A repeat transvaginal ultrasound conducted two weeks later revealed a normal endometrial appearance (Figure 3).

## Discussion

This case highlights a rare but important complication following surgical management of miscarriage, hematometra. While hematometra is more commonly associated with congenital anomalies, cervical stenosis, or surgical scarring, it remains an under-recognized cause of post-procedural pelvic pain and should be considered in the differential diagnosis, particularly in the early postoperative period. Hematometra after uterine instrumentation, such as suction evacuation or dilation and curettage, is reported in approximately 0.1%-1% of the cases [1].

This patient's presentation within 48 hours of surgical evacuation with severe, contraction-like pelvic pain, urinary and bowel discomfort, and localized uterine tenderness initially raised concerns for endometritis or uterine perforation, both of which are established complications of surgical uterine evacuation. Notably, her general observations remained stable and laboratory findings were non-specific apart from a mildly elevated CRP, which may reflect a reactive inflammatory response rather than overt infection. The symptoms of hematometra include pelvic pressure or pain, urinary retention, and tenesmus [3]. These symptoms were seen in our patient as well. Transvaginal ultrasonography proved critical in clarifying the diagnosis, revealing a distended endometrial cavity containing hypoechoic material consistent with hematometra, without signs of retained products of conception (RPOC) or abnormal vascularity. The absence of RPOC likely ruled out ongoing trophoblastic tissue activity, reducing the risk of persistent bleeding or gestational trophoblastic disease. Contrast-enhanced computed tomography (CECT) and magnetic resonance imaging (MRI) of the pelvis can also be used to aid the diagnosis [4]. In our case transvaginal sonography confirmed the diagnosis. Hematometra formation in this case may be attributed to cervical stenosis or inadequate cervical dilation, potentially exacerbated by post-surgical inflammation or rapid cervical closure. The patient’s need for internal os dilation during the second procedure supports this hypothesis. Cervical stenosis leading to blood pooling post-evacuation is well described. Mallick et al. refer to this as “post-abortal syndrome,” with delayed presentation of colicky pain and hematometra in early post-procedure weeks [1]. Though rare, iatrogenic cervical stenosis can develop after uterine instrumentation and is a recognized cause of outflow obstruction leading to hematometra. Reddy et al. described a case of hematometra three years following laparoscopic myomectomy [4]. Sharhaki et al. described cases of hematometra following caesarean section [2]. Kaur et al. reported recurrent hematometra formation as a rare complication following cesarean delivery, and the patient underwent hysteroscopy with cervical dilatation, which served both diagnostic and therapeutic purposes [5]. A similar case was reported by Yared et al. in which the patient had multiple abortions and underwent suction evacuations for hematometra [6]. Hematometra can develop after a ceserean section or abortion. In teenagers, it's most often due to congenital anomalies [6]. Hematometra following abortion is mostly due to retained products of conception or acquired cervical stenosis [6]. Another study mentioned hematometra with retained products of conception, where the patient presented with chronic abdominal pain and bleeding [7]. Murakami et al. reported a rare case of hematometra in ceserean scar defect and the patient required emergency hysterectomy [8]. Surgical management of hematometra is often required, especially where conservative management has not provided relief [6]. Yared et al. described giving oxytocin 10 units following the surgical procedure [6]. In our case, no uterotonics were given as her bleeding was controlled. Uno et al. described a rare case of spontaneously perforated pyometra, highlighting the importance of timely diagnosis and management to prevent catastrophic outcomes [9]. Ohara described an acute onset haematometra associated with endometritis and cervical stenosis following a suction evacuation, which emphasized the need for careful monitoring and potential intervention to prevent such complications [10]. The resolution of symptoms following re-evacuation and the return to a normal endometrial appearance on follow-up imaging supports the effectiveness of timely intervention. This case underscores the importance of maintaining a high index of suspicion for hematometra in patients with persistent or worsening pelvic pain after uterine evacuation, particularly when more serious complications like perforation are ruled out. Although the reviewed literature discusses hematometra arising from various etiologies, the underlying cause in most cases is acquired, primarily due to cervical stenosis secondary to infection, inflammation, or surgical trauma. These factors can lead to obstruction of menstrual blood outflow, resulting in the accumulation of blood within the uterine cavity. Infection and inflammation may cause scarring and narrowing of the cervical canal, while surgical interventions such as dilation and curettage, cesarean sections, or other uterine procedures can contribute to structural changes that predispose patients to hematometra. There is also a notable lack of literature exploring the relationship between hematometra and assisted reproductive technologies, particularly in the context of same-sex couples undergoing in vitro fertilization (IVF).

Furthermore, this case serves as a reminder of the unique psychosocial and clinical considerations in patients who conceive through assisted reproductive technologies (ART), such as IVF. Emotional investment in the pregnancy, often higher in such cases, may influence how symptoms are perceived and reported, underscoring the need for compassionate and thorough evaluation.

## Conclusions

This case highlights hematometra as a rare but important complication following surgical management of miscarriage. While often overlooked, hematometra should be considered in the differential diagnosis of acute pelvic pain after uterine evacuation, particularly in the absence of significant vaginal bleeding. Early recognition through transvaginal ultrasonography and timely intervention can lead to prompt symptom resolution and prevent further morbidity. Additionally, special attention should be given to the emotional and clinical complexities in patients who have conceived through assisted reproductive technologies, emphasizing the importance of a compassionate and comprehensive approach to post-procedural care.
